# Alzheimer's Disease Begins Years Before Symptoms: Alzheimer's Association Workgroup

**DOI:** 10.1002/alz70856_107830

**Published:** 2026-01-09

**Authors:** Reisa A. Sperling, Alzheimer's Association Revised Criteria Workgroup

**Affiliations:** ^1^ Center for Alzheimer's Research and Treatment, Department of Neurology, Brigham and Women's Hospital, Harvard Medical School, Boston, MA, USA

## Abstract

**Background:**

The major differences between the Alzheimer's Association (AA) and International Working Group (IWG) positions are focused on cognitively unimpaired (CU) individuals with abnormal AD biomarkers. Here we aim to explicate the Alzheimer's Association's (AA) workgroup position on the preclinical stage of Alzheimer's disease (AD).

**Method:**

The AA Workgroup defines AD as the pathophysiological process in the brain, which begins with accumulation of amyloid and tau pathology more than a decade prior to clinically evident impairment. We acknowledge that some individuals with abnormal AD biomarkers will not progress to cognitive impairment within their lifetime, and explicitly recommend against testing or disclosing test results to asymptomatic people outside of a research setting at this time. The recognition, however, that AD is a continuum of disease that includes the preclinical phase is essential for developing treatments that may be most efficacious prior to clinical impairment, when there is already substantial neuronal damage.

**Result:**

The requirement for clinical symptoms to diagnose AD is inconsistent with the 21st century definitions of almost all other chronic diseases, including cardiovascular disease, cancer, and diabetes. It is very reasonable to discuss “risk” in terms of likelihood of developing impairment over a given time frame ‐ but this is risk of manifesting symptoms related to the underlying disease. A person cannot simultaneously have a disease and be at risk for that disease. If clinical symptoms are required to define disease, it seems inconsistent that the IWG would classify an asymptomatic autosomal dominant carrier as “presymptomatic disease”. The level of biomarker abnormality is relevant to the timeframe of progression, as >50% of CU with high amyloid and tau progress to MCI/dementia within 5 years but waiting for biomarker “certainty” of imminent clinical progression to identify “disease” runs the risk of intervening too late.

**Conclusion:**

Both IWG and AA groups carefully consider the potential risks of “overdiagnosis” and stigma raised by using the “A” word. Similar to the revolution that occurred in cancer, the AA prioritizes research in early identification of disease and intervention as an important step towards reducing that stigma.

